# PReS-FINAL-2291: Activation of TLR pathway JSLE derived neutrophil extracellular traps

**DOI:** 10.1186/1546-0096-11-S2-P281

**Published:** 2013-12-05

**Authors:** C Thorbinson, A Midgley, MW Beresford

**Affiliations:** 1 Institute of Child Health, University of Liverpool, Liverpool, UK

## Introduction

Juvenile Systemic Lupus Erythematous (JSLE) is characterised by auto-antibody production directed against nuclear auto-antigens. Toll-like receptors (TLRs) are pattern recognition receptors of the innate immune system responsible for initiating an immune response against invading pathogens. TLR 3, 7-9 have been studied in SLE due to their unique ability to detect nuclear antigen. Their expression is increased in JSLE and significantly associated with disease activity and anti-dsDNA titres. Upon recognition of an extracellular pathogen, neutrophils may release neutrophil extracellular traps (NETs) containing antimicrobial peptides to capture and neutralise pathogens. Nuclear material including DNA and histones comprise the major structural components of NETs and may act as a source of nuclear auto-antigen in SLE. The mechanism by which NETs may induce an auto-inflammatory response has not been elucidated. Our hypothesis is that neutrophil NETs are a source of nuclear auto-antigen in JSLE being detected through the TLR pathway leading to an auto-inflammatory response.

## Objectives

To investigate whether NETs are able to activate the TLR pathway, using pIRAK1, a signalling protein specific to the TLR pathway

## Methods

Neutrophils were isolated from children with JSLE and paediatric & adult non-inflammatory controls and were either left unstimulated or incubated with 100ng IFN-alpha for 2 hours. Induction of NETs was visualised using confocal microscopy. Extracellular DNA was measured using the Quant-iT Picogreen assay (Invitrogen, Carlsbad, CA).

PBMCs were isolated from healthy adult controls and either left unstimulated or induced with LPS, TLR7/TLR9 agonist, as a positive control, or NETs derived from JSLE, paediatric or adult control neutrophils with +/- IFN-α for 30 minutes; cell protein was then extracted. pIRAK1 protein expression was determined by Western blot and normalised to β-actin expression.

## Results

Cells incubated with LPS (×1.4 fold), TLR 7 (×1.7) & 9 (×1.7) agonists and NETs showed increased pIRAK protein expression as compared to unstimulated PBMCs. PBMCs incubated with NETs containing higher concentrations of dsDNA showed a greater fold increase in pIRAK1 protein expression. Increased expression did not seem to be influenced by origin of NET.

## Conclusion

Neutrophil NETosis has been proposed as a potential mechanism for auto-antigen exposure in SLE and has been shown to be dysregulated in lupus and correlate with lupus nephritis. Here we have shown that NETs are capable of activating the TLR 7/9 pathway and suggest that this is one mechanism by which an autoimmune response is driven. We have shown this to occur in a dose dependent manner to dsDNA. This data adds to the growing body of evidence supporting the role of TLRs in JSLE and the potential benefit of using TLR inhibition therapy in JSLE.

## Disclosure of interest

None declared.

**Table 1:** 

Cell Stimulation	dsDNA Concentration (ng)	Fold increase in pIRAK1 protein expression
Unstimulated PBMC	NA	0
Paediatric Control NET - IFN-α + IFN-α	0.2 33.7	1.9 2.1
JSLE NET - IFN-α + IFN-α	12.2 32.3	1.9 2.1
Adult Control NET + IFN-α	111.8	2.9

